# Interment Private

**Published:** 1884-07

**Authors:** Ben. H. Pratt


### THE BISTOURY.

Vol. XX. ELMIRA, N. Y., JULY, 1884. No. 2.

### Contributions.

### "INTERMENT PRIVATE."

BEN. H. PRATT.

Now and then one is permitted to note the attachment of the above phrase to the ordinary obituary notice. It is rarely so affixed, it is true, but the rarity indicates a tendency in the direction of a very much needed reform, a tendency that inspires a hope that the time may not be very far distant when public funerals will be but vulgar, unbecoming, ostentatious exceptions to the funereal proprieties, a light in which they are viewed by many even at this early day, and "interment private" will be understood as a matter of course, not needing to be appended to the notice of death, for the notice of burial will then never be made at all. It is hoped that the time is not far off when the grief stricken will not be called upon to have their emotions inspected, and their funereal manner and apparel inventoried, and their measure of grief estimated and rehearsed in the marts of social gossip. May the time speedily come when one will not be compelled to parade his sorrow and trumpet his lamentations; when the sorely wounded heart that has but now ceased to bleed, and has entered upon the healing process, shall be allowed to continue under the merciful and efficacious ministering of time, and not be subjected to an opening of its wounds afresh through the barbarously cruel revival of all the tender memories of the deceased during that modern abomination, a public funeral; when mourning friends will be permitted to indulge their grief apart from the prying eyes and the embarrassing observation of any who may be bold enough and curious enough and indelicate enough to intrude upon emotions that are sacred; when, in a word, the home into which death shall have come may be absolutely private, except so far as outer aid may be necessary, or may be invoked for the sake of withdrawing the mind from a contemplation of its sorrow, and directing it toward a proper cheerfulness.

Under the present system of conducting funerals it is difficult to say which occasion, the hour of death or the hour of the funeral, is the more to be dreaded, the more harrowing to the soul. It seems the acme of cruelty to thus duplicate the mourners' grief. Poignant as may be the pang of affliction in the knowledge of the death of a loved one, it is sadly, wickedly intensified by the accompaniments of the modern funeral. The funeral is always the dread of the stricken. True grief is retiring. It would hide its face from the gaze of any save the near and dear, and begs to be let alone. The presence of any except the closest friends at such a time is torture. To have a great sorrow and to feel that it may not be expressed; to realize that the emotion demanding vent must be suppressed within the bounds of social propriety; to be compelled to think how one must dress, and act, and endure the popular gaze, while the heart is in the depths of woe for the lost one; to know that men and women and children whom you know, but for whose company you do not care at such a time, will flock into your presence to mark your grief and remark upon your demeanor under that grief; to feel that the minister, nine times out of ten not only an unwilling celebrator of the funereal rite, but actually dreading it, must come and say things that cannot console you one particle, but will only reopen afresh the wounds you have received; to feel that singers will come, and with strains, never so sweetly rendered, will only renew and intensify your emotion; to be sure that everything will be done with the best intention, that of consolation, but with the worst of results, that of reviving the keenness of the loss you have sustained ; to know that under this pressure you are presumed to be comforted while you are actually being agonized ; these are simply humane cruelties, the keenness of which none can know until they have endured them.

The origin of the funeral ceremonial was doubtless a humane instinct. It is natural that the heart of every friend of the afflicted should be touched with pity for him. It is natural to wish to visit and to strive to comfort him. It is natural, too, for one to wish to assist the afflicted in every possible way, and to relieve the sorrowing of all care and responsibility under the great stress of grief. It is natural, too, for the friend to wish to show you, the mourner, how much he too is concerned for the death of your dear one, and he becomes profusely officious in the desire to prove his interest in your sorrow. There is no intention, perhaps, to pry into your affliction, and no other desire than to assuage your grief. But the act is a mistaken one. Under the impression that the stricken is somewhat comforted by the presence of friends at the funeral of one whom he is about to lay away forever, friends flock in and say and do and look things that are sadly at variance with the situation. The result is always annoying, sometimes harrowing. often atrocious.

How much better it would be if, after the death of a member of the household, no one were to enter therein without a summons from the bereft ; if the living could be permitted to vent their grief unchecked by the presence of others ; if there need be no preparations for a formal ceremony and a solemn and awe-inspiring gathering of sad faced people to remind one, all over again, of the terrible loss one has sustained ; if the body of the loved one might be allowed to remain until the relatives were reconciled to the burial, and then have that duty performed without announcement. without parade and ceremony, without soul harrowing reminders of the virtues and excellencies of the departed, and above all without being compelled to make such demonstrations of grief as nature compels, in the presence of a crowd of people, many of whom are present as a matter of duty or through a morbid curiosity.

The entire system of public funerals is wrong. It is even more indelicate than it would be to call into one's home a host of one's friends to witness a birth. The principle is a mistaken one. Words do not console at such a time. The preachers who officiate at funerals have long since arrived at this conclusion, and now and then there arises a clergyman who dares to assert his unbelief in the propriety of funeral ceremonies. Some positively decline invitations to speak on such occasions ; others consent to merely read a portion of scripture, make a prayer and suffer a hymn to be sung ; others still, claim that a few words of ritual at the grave are all that the occasion demands, and all they will consent to utter.

Aside from the misery that a public funeral brings to the family of the deceased is the unnecessary awe which is entailed upon an entire neighborhood. The undertaker drives to the door with his casket and box, with his ice chest, with his casket stands, with his camp chairs and other paraphernalia of funeral necessities. The hearse and the carriages come and for hours block the highway and advertise the place of mourning. Solemn looking people arrive, speak in whispers, stand uncomfortably without the door or gather ruefully within the house. There is a demonstration without any fitness whatever. It is unnecessary, it is distasteful, it is dreaded, it is wrong. There is no sense in the whole proceeding. It is but a contemptible observance of a custom that would be "more honored in the breach than the observance." It makes the unhappy unhappier, and only contributes to the purse of the undertaker and the liveryman. It is complied with merely because custom says that this is an ordeal which all mourners must pass through if they would conform to the social usage.

Thank heaven there are some people, now-a-days, who have the temerity to attach to the published notice of a death, "interment private." Of course it excites remark. Of course it arouses wonder. Of course it causes a brief season of gossip, and much speculation as to why the public is not invited. or at least allowed, to assemble and gloat over the grief of the bereaved ones. And yet no one who considers the matter can give any good reason why he should be called upon to intrude himself upon the privacy of people when privacy is demanded with more reason than at any other period of existence. If there is any time when one should be let alone ; when one should be regarded as beyond the reach of any human solace ; when he should be allowed to contend with his grief without the extraneous assistance of others, it is in the presence of his dead. Common humanity, common politeness, common respect, common decency demand this.

Public funerals, with their show of funereal paraphernalia, with their gathering in of curious, long-visaged, solemn people ; with their wordy clergymen : with their doleful singers ; with their crape decked pall bearers; with their public procession from the chamber of mourning to the carriages in waiting ; with their solemn journey to the church; with more heart rending words from the minister and more dole-begetting music ; with more parading up and down the aisles of the church ; with the slow journey to the burial ground ; with another parade of the mourners about the open grave ; with more saddening words from the men whose office is to comfort and not to torture the soul ; with that barbarous, dreadful, blood curdling, outrageous rattle of gravel upon the coffin lid, to the atrocious accompaniment of "Earth to earth, ashes to ashes, dust to dust," that is an almost invariable portion of the horid ceremonial at the grave ; with the conventional standing of the mourners about the pit while the diggers shovel back the earth over the form of the one just laid away ; with all this empty, useless, cruel and damnable ceremonial ; public funerals are simply schemes of human torture, and the sooner the whole system is abolished the better. The cruelest act we can perform is to intrude our presence upon those who are in agony over the loss of their friends. It must needs be that dear ones die, and it is equally necessary that they be borne away out of sight. Is not the agony sufficiently acute without intensifying it by parading it before the gaze of the multitude ? Therefore the tendency in the direction of "interment private" is hailed as an encouraging one and productive of a hope that the time is coming when all interments will be such, and the mourner be allowed to vent his grief and relieve his soul without the consciousness that he is being stared at; without having to worry as to how he may appear to his fellowmen in his grief. Funerals should be purely family gatherings, and the fewer present the better.

By and by there will be few interments, few errands to the cemeteries, few cemetries, in fact. Cremation will in time become necessary, but it is to be hoped that it will be popular before the necessity arises. The dead will then be but temporarily borne from the sight of friends and the remains, inurned, returned to the home to be placed in the home's receptacle for the departed. May that time swiftly arrive. It will do more to abolish the cruel custom of parading the grief of the mourning ones than anything else. The dreadful thought, with most people, is the desertion of the bodies of loved ones in the cold earth of the lonely church yard. When mankind shall have become educated to the idea that the present ashes of the beloved dead are preferable to their forever lost and possibly scattered dust, the more easily and the less shudderingly will mankind become reconciled to death.

